# Gastric Digestion

**Published:** 1890-02

**Authors:** 


### Gastric Digestion.

At the meeting of the Philadelphia County Medical Society, October 9, 1889, Dr. L. Wolff read a paper on the Chemistry of Gastric Digestion, in which he said:

The gastric secretion which causes the chemical change of protein bodies into peptone has been closely studied by C. Schmidt, who determined the total chlorine therein, together with all the bases, such as potassium, sodium, calcium, magnesia, ammonium, and iron, and after accounting for the saturation of these had a residue of free hydrochloric acid left which amounted to about 2.5 to 4 grammes in one litre of the secretion. Although the pure hydrochloric acid has no peptonizing action, it alone can make pepsin display such a function, while again neutral pepsin has no digestive power unless coupled with this acid. While it is thus evident that the hydrochloric acid plays an important part in gastric digestion, it has an equally valuable property by acting as an antiseptic and germicide over the ingesta when present in a free state. Miguel ascertained that 0.2 to 0.3 grammes of a mineral acid sufficed to preserve 100 c.c. meat broth from sepsis, after Siebert had shown that 0.5 per cent. HCl prevented chopped meat effectually from decay. As had been proven in Hoppe-Seyler's laboratory that there existed in the normal gastric secretion 0.3 per cent. HCl, it would readily appear how the normal amount of HCl in the gastric juice will prevent the development of sepsis in the food bolus, while the presence of less than the normal amount will permit some ferment action, and the total absence will result in fermentation with production of organic acids which will not only not serve to develop the digestive power of pepsin, but will retard and prevent it.

The question of the production of such a strong mineral acid by the alkaline tissues of the stomach and from the alkaline blood can be only explained by the presence of chlorides in the blood, and the decomposition thereof in the secretory apparatus, with the elective osmosis of the ovoid cells which separate the acid to the interior of the gland while it sends back more alkaline blood to the circulation. It is a fact, well established, that weaker acids may replace stronger ones, and it appears a fair deduction that the alkaline hydrocarbonates of the blood become, under the influence of the secreting cell, neutral carbonates, by displacing from the chlorides the chlorine, which unites with the hydrogen so liberated to form hydrochloric acid. As to the intimate function of the secretory apparatus in the production of HCl, little is known, though it seems proven to a certainty by Heidenhain that the ovoid cells of the tubules of the peptic glands secrete the acid, for on the tubules of the pyloric glands no ovid cells are found, and no HCl is secreted by them, although the secretion of pepsin by them is readily shown by the digestive test with addition of hydrochloric acid.

